# Knowledge, attitude and disinformation regarding vaccination and immunization practices among healthcare workers of a third-level paediatric hospital

**DOI:** 10.1186/s13052-019-0684-0

**Published:** 2019-08-19

**Authors:** Carlotta Tomboloni, Chiara Tersigni, Maurizio de Martino, Donata Dini, José Rafael González-López, Filippo Festini, Stella Neri, Daniele Ciofi

**Affiliations:** 1 Oxford University Hospital NHS Foundation Trust, Collaborator of the Italian Science Society of Paediatric Nursing, Oxford, UK; 2 Department of Health Sciences, University of Florence, Florence, Italy; 3 Department of Health Sciences, Anna Meyer Children’s Hospital, University of Florence, Florence, Italy; 4 Anna Meyer Children’s Hospital, Florence, Italy; 5 Faculty of Nursing, Physiotherapy and Podiatry, Nursing Department, Universidad de Sevilla, Seville, Spain; 6 Azienda USL Toscana Centro, Florence, Italy

**Keywords:** Vaccination, Knowledge, Attitude, Skills, Disinformation, Immunisation, Practices, Healthcare workers, Paediatric hospital

## Abstract

**Background:**

Vaccination represents one of the most effective means of preventing infections for the population and for the public health in general. Recently there has been a decline in vaccinations, also among healthcare workers (HCWs). The aim of the study is to detect the knowledge, skills, attitudes and barriers of HCWs regarding vaccinations in a tertiary children’s hospital in order to support clinical management in immunisation practices.

**Methods:**

An observational study was conducted on 255 subjects over a period of 8 months. The 31-item questionnaire considered profession, level of instruction and different ages. It included questions taken from a questionnaire used for a Canadian research and one used by the Bellinzona hospital. A 4-point Likert scale and closed-ended questions were used. A confidence interval of 95%, *p* value ≤ 0.05, Chi-square, ANOVA and the Kruskal-Wallis test were considered.

**Results:**

In the last 5 years less than one third of the sample were vaccinated against flu. 77.8% (n.130) of nurses and 45.8% (n.19) of doctors were not vaccinated (*p* < 0.0001).

As for risk perception, 51.5% of nurses and 90.6% of doctors believe that their risk of contracting influenza is greater than that of the general population.

In relation to the injection site, in all the age ranges there was a high level of knowledge except for those aged over 61 who responded incorrectly. Doctors were more prepared (*p* < 0.0001).

50% of the sample used internet only as a source of information for vaccines. Generally, scientific sources were used infrequently. The higher the education level, the more frequent the utilisation of trustworthy scientific resources and literature. (*p* = 0.0002).

**Conclusions:**

In line with the attitude observed in recent years, nurses are not inclined to get vaccinated themselves although they agree to having their children vaccinated. HCWs have a good level of knowledge about vaccines and immunisation practices.

With the nurses we found that the higher the education level, the greater the knowledge about vaccines which leads to the conclusion that low levels of adherence are not due to a lack of knowledge, but rather, to a low perception of risks. Hence the need to strengthen the vaccination strategies inside the companies.

## Background

Vaccination represents one of the most effective and secure means of primary prevention of infectious diseases for people and the for the public health in general. This practice gives direct benefits from vaccines and a form of indirect protection for individuals who are not immune (herd or social immunity). Thanks to the immunisation practice it has been possible to revolutionise the history of medicine, reducing mortality and morbidity and profoundly changing the epidemiology of infectious diseases [1].

The practice and use of vaccinations have led to the disappearance of smallpox for example. The World Health Organisation declared human beings smallpox-free on 8 May 1980 and poliomyelitis-free in Europe in June 2002 [1].

Compulsory vaccinations together with the flu vaccination are included in the basic levels of medical assistance and actively offered all over Italy even if the levels of coverage in different geographic areas are heterogeneous [1].

In Italy, vaccination policies have been characterised by a strong heterogeneity; the same vaccination could be offered for free to all new-born babies in one region, or only to subjects at risk in other regions. Differences can even be found within the same region among single local health units [1, 2].

In 2007, the Veneto region suspended compulsory vaccinations and introduced a monitoring system for vaccine coverage [3]. This tendency, followed by other regions, plus the decrease in adherence to vaccinations and the re-emergence of several paediatric-age diseases have led the Italian government to introduce Legislative Decree 73/2017 [4].

The new law has established 12 compulsory vaccinations for 0–16 year-old children with fines of up to 5,000 Euro for defaulters.

Concomitantly, a low level of information has been observed among professional healthcare workers (HCWs), giving rise to strong scepticism about the efficacy and safety of certain vaccinations which can lead to widespread under-utilisation. This attitude of mistrust manifested itself clearly after the recent influenza pandemic, during which healthcare workers showed very low support of vaccination campaigns although flu vaccination is recommended for all at-risk groups, including the HCWs themselves [1–7].

The issue of vaccination among HCWs is undoubtedly important for the protection of the health not only of the workers themselves, but also of the whole community.

Not all HCWs are in favour of vaccinations and as proof of this we can mention the innumerable websites and HCW associations which are actively engaged against vaccines. In fact, the rate of support for some vaccinations among HCWs never reaches 100% [8, 9]. .Among the reasons that induce healthcare workers not to get vaccinated are the strong uncertainties about the actual safety and efficacy of the vaccine itself, fear related to adverse reactions, fear of injection-related pain, fear that multiple injections at one time could be too much for the immune system, the belief that it is not necessary to be vaccinated, and finally, the organisational difficulties in obtaining the vaccination [10–12].

Many American studies have shown that although the rate remains low, more than 50% of HCWs are in favour of vaccinations, especially against flu [13].

In support of the importance of vaccinations, several studies report the reasons which include the professional responsibility to protect themselves, their patients and colleagues, but also family members, as well as the ease of access to vaccines and their gratuity [12].

In general, however, in recent years there has been a decline in the support rates of vaccinations among citizens and HCWs alike [14].

However, some American studies disagree with the above-mentioned statements, probably supported by the increase in mandatory vaccination policies in their hospitals [15].

In recent years, in parallel with this imbalance in the support rate of vaccinations, several studies have evaluated the attitude, knowledge and immunisation practice of HCWs [12, 13, 16–18].

In addition to the alarming data about the HCW rates of support of vaccinations, the most worrying data is represented by their alleged inclination not to get vaccinated, which could discourage the choice of the citizens themselves [19].

For this reason, it is necessary to promote the immunisation practice as well as for HCWs to acquire information for raising awareness of the problem [20].

Hence the research project was initiated on “Knowledge, attitude and disinformation regarding vaccination and immunisation practices among HCWs in a third-level paediatric hospital.”

The survey is designed to detect knowledge, skills, attitudes and barriers regarding vaccinations of HCWs at the “Anna Meyer” Children’s Hospital of Florence (CHoF) in order to support clinical management in decision-making capable of positively affecting the immunisation practice.

## Methods

### Design and setting

The project was an observational and correlation research about knowledge, attitudes and disinformation regarding vaccination and immunisation practices among HCWs at the Children’s Hospital of Florence.

The hospital employs 1,000 HCWs, 540 nurses and 300 doctors, plus technicians, administrative staff and students. The hospital comprises 65 inpatient and outpatient services, with 250 inpatient beds.

### Population

HCWs with permanent and temporary contracts since the beginning of the research (1/1/2015), nurses, paediatricians, technicians, radiologists, pharmacists and student nurses. Healthcare assistants and administrative staff were excluded.

From 860 units the sample size was calculated using the predetermination of a sample size of 20%, a confidence interval of 95% and *P* value < 0.05. At the end of the research 255 subjects had been included.

### Period of time

After consent was obtained from the General Directorate (not necessary from the ethics committee as no patients were involved), the study took 4 months to reach the sample size and a further 4 to be completed, for a total of 8 months from the beginning of the research (1/1/2015).

### Assessment

Data were joined anonymously with the help of EXCEL and EPINFO programmes. Confidence interval 95%, *p* value ≤ 0,05. Mode mean, and median were evaluated, and Chi-square, ANOVA and the Kruskal-Wallis test were considered.

### Statistical methodology and outcome

The questionnaire was divided into three parts with a total of 31 questions.

The first part considered the profession, level of education, gender, age and length of service. Answers for age and length of service were arranged into categories of 10 years each.

The second part included questions taken from another questionnaire which had been used for a Canadian research about vaccines, and implemented with new questions. The 4-point Likert scale was been used, 1 for “Strongly disagree”, 2 for “Not fully agree”, 3 for “Pretty much agree” and 4 for “Strongly agree”.

The third part was based on a questionnaire used by the Bellinzona hospital for a previous research about flu jab with closed-ended questions.

## Results

The study analysed a sample of 255 units.

12.2% (n.31) males and 76.8% (n.196) females. 11% (n.28) did not state the gender.

66.3% (n.169) were registered nurses and registered paediatric nurses, 2.7% (n.7) were pharmacists. The medical doctors who completed the questionnaire were 20.8% (n.53), and 7.1 (n.18) technicians and 3.1% (n.8) nursing students also participated in the study.

As regards the age, 31.8% (n.81) were aged between 20 and 30, 28.6% (n.73) between 41 and 50, 27.8% (n.71) between 31 and 40, 10,6% (n.27) between 51 and 60, and 1.2% (n.3) were over 61 years.

As for length of service, 52.9% (n.135) had worked for 0 to 10 years, 22.8% (n.58) for 11 to 20 years, 18% (n.46) for 21 to 30 years, and 6.3% (n.16) for 31 to 40 years.

We asked the sample if they knew what herd immunity is. Only 248 subjects answered the question. Table 1 shows the results.

**Table 1:** Knowledge of herd immunity effect among health care workers

Do you know what herd immunity is?			
	Yes	No	Total
Total	65.7% (n.163)	34.3% (n.85)	100% (n.248)
Nurses	54.9% (n.90)	45.,1% (n.74)	100% (n.164)
Paediatricians	98.1% (n.51)	1.9% (n.1)	100% (n.52)
Pharmacists	66.7% (n.4)	33.3% (n.2)	100% (n.6)
Radiologists	72.2% (n.13)	27.8% (n.5)	100% (n.18)
Students	62.5% (n.5)	37.5% (n.3)	100% (n.8)

More specifically, for nurses with different degree levels, the higher the education level, the greater the knowledge. 39.8% (n.29) of nurses with a diploma answered yes, 60.2% (n.44) answered no. 64.3%, (n.45) of nurses with a bachelor degree answered yes, 35.7% (n.25) answered no. 71.4% (n.5) of nurses with a master answered yes, 28.6% (n.2) answered no. 80% (n.8) of nurses with a post graduate degree answered yes, 20% (n.2) no. 100% (n.1) of Nurses with a PhD answered yes.

We also investigated the level of knowledge of the sample regarding the site of the vaccine injection for children under 1 year of age. (Table 2). For subjects aged between 20 and 30, 75.3% (n.61) answered correctly, 24.7% (n.20) incorrectly, for those aged between 31 and 40, 87.3% (n.62) answered correctly and 12.7% (n.9) incorrectly, for those between 41 and 50, 84.9% (n.62) answered correctly and 15,1% (n.11) incorrectly, and for those between 51 and 60, 63% (n.17) answered correctly and 37% (n.10) incorrectly. For subjects aged over 61, 33.3% (n.1) answered correctly and 66.7% (n.2) incorrectly.

**Table 2:** Level of Knowledge about vaccine injection site for a child under 1year among health care workers

What is the vaccine injection site for a child under 1 year?			
	Correct	Incorrect	Total
Nurses	79.9% (n.135)	20.1% (n.34)	100% (n.169)
Paediatricians	100% (n.53)	0%	100% (n.53)
Students	25% (n.2)	75% (n.6)	100% (n.8)
Radiologists	61.1% (n.11)	38.9% (n.7)	100% (n.18)
Pharmacists	28.6% (n.2)	71.4% (n.5)	100% (n.7)

correct answer: anterolateral thigh

As regards vaccinating their own children, 249 units answered the question. 1.6% (n.4) did not agree while 73% (n.183) agreed. The other 25.4% did not answer the question. Following this positive inclination to get their children vaccinated, we investigated whether HCWs had had flu jabs themselves over the last 5 years. Table 3 shows the results.

**Table 3:** Percentage of adherence to the flu jab among health care workers

Did you have a flu jab in the last 5 years?				
	No	Once	Twice or More	Total
Nurses	77.8% (n.130)	14.4% (n.24)	7.8% (n.13)	100% (n.167)
Paediatricians	35.8% (n.19)	34% (n.18)	30.2% (n.16)	100% (n.53)
Pharmacists	85.7% (n.6)	14.3% (n.1)	0	100% (n.7)
Radiologists	72.2% (n.13)	22.2% (n.4)	5.6% (n.1)	100% (n.18)
Students	62.5% (n.5)	37.5% (n.3)	0	100% (n.8)
Total	68.3% (n.173)	19.8% (n.50)	11.9% (n.30)	100% (n.253)

As for the source of information about vaccines, 12.4% (n.31) indicated the internet, 13.5% (n. 34) books and scientific publications, 8% (n.20) colleagues, and 13.9% (n.35) the family paediatrician. 52.2% (n.131) stated they use multiple sources with no preferences between any of them.

Table 4 in particular, shows the source of information about vaccines among nurses with different degree levels.

**Table 4:** Sources of information for vaccines used among nurses with different education level

Which source of information do you use?						
	Internet	Scientific Publication	Colleagues	Paediatricians	Multiple Answers	Total
Diploma	20.8% (n.15)	9.7% (n.7)	1.4% (n.1)	20.8% (n.15)	47.3% (n.34)	100% (n.72)
PhD	0	0	0	0	100% (n.1)	100% (.1)
Post Graduate	0	14.3% (n.1)	14.3% (n.1)	0	71.4% (n.5)	100% (n.7)
Bachelor	12.3% (n.9)	2.7% (n.2)	17.8% (n.13)	16.4% (n.12)	50.8% (n.37)	100% (n.73)
Master	0	0	0	20% (n.2)	80% (n.8)	100% (n.10)

We asked the sample if, as HCWs they think it is important to offer mandatory vaccinations. 254 units answered the question: “As a HCW, it is important to offer mandatory vaccination”. 1.2% (n.3) strongly disagree, 1.6% (n.4) do not fully agree, 19.6% (n.50) pretty much agree and 77.6% (n.197) strongly agree.

We also investigated whether the sample believed in the benefits of vaccines. 254 units answered the question: “Vaccine brings more benefits than risks”. 1.2% (n.3) strongly disagreed, 2.8% (n.7) did not fully agree, 32.2% (n.82) pretty much agreed and 63.8% (n.162) strongly agreed.

Following this result, we also investigated the use of alternatives to vaccination among the sample. Only 249 units answered the question. 98.4% (n. 245) do not use any, 0.4% (n.1) answered yes without specifying, 0.4% (n.1) cited vitamins to increase immunity against flu, 0.4% (n.1) mentioned homeopathy and another 0.4% (n.1) mentioned holistic disciplines. Table 5 shows the results specifically for nurses with different degree levels.

**Table 5:** Alternatives to vaccination used among nurses with different education level

Are there any alternatives to vaccination?						
	No	Yes	Drugs to increase immunity	Homeopathy	Holistic Disciplines	Total
Diploma	95.8% (n.69)	0	1.4% (n.1)	1.4% (n.1)	1.4% (n.1)	100% (n.72)
Post graduate	100% (n.7)	0	0	0	0	100% (n.7)
PhD	100% (n.1)	0	0	0	0	100% (n.1)
Bachelor	98.6% (n.71)	1.4% (n.1)	0	0	0	100% (n.72)
Master	100% (n.10)	0	0	0	0	100% (n.10)

We believed it was very important to understand the HCW’s perception of the risk of influenza. We asked them how much the risk for them getting influenza is, compared to the normal population.

38.2% (n.63) of nurses believed the risk is higher, 51.5% (n.85) the same and 10.3% (n.17) lower. 57.1% (n.4) of pharmacists believed the risk is higher, 42.9% (n.3) the same. 90.6% (n.48) of paediatricians believed the risk is higher, 7.5% (n.4) the same and 1.9% (n.1) lower. 62.5% (n. 5) of students believed the risk is higher, 37.5% (n.3) the same. 44.4% (n. 8) of radiologists believed the risk is higher, 50% (n.9) the same and 5.6% (n.1) lower.

Regarding adverse vaccine reactions, we asked the sample which one they were most worried about. Table 6 shows the results. Two subjects did not answer the question.

**Table 6:** Most worrying adverse vaccine reactions among health care workers

Which of the following adverse vaccine reactions are you most worried about?	
Neurological adverse reactions (e.g. Guillan Barrè)	13.3% (n.34)
Severe allergic reactions	9% (n.23)
Fatigue and weakness	5.1% (n.13)
None of them	38.4% (n.98)
Multiple answers	34.2% (n.85)
Total	100% (n.253)

Finally, we investigated which strategies the units think would work better to increase the flu jab rate. Two hundred and forty-four subjects answered the question. 38.5% (n. 94) proposed a personal invitation letter, 13.9% (n.34) think the use of link nurses would be useful in each ward, 13.1% (n. 32) proposed the use of posters to raise awareness, 5.7% (n.14) proposed money incentives, and 11.1% (n.27) proposed mandatory vaccination for all employees. The other 17.5% (n. 43) proposed multiple options.

## Discussion

### Knowledge

The benefit of achieving important goals such as a reduction in the prevalence of some vaccine preventable diseases, may be found not only in appropriate and satisfactory vaccination coverage, but also in the chance to exploit herd immunity in cases where is not possible to reach a 100% vaccination coverage [1].

Herd immunity is a form of indirect protection from infectious disease that occurs when a large percentage of a population are vaccinated, thus providing a measure of protection for individuals who are not immune.

In other words, vaccinated units not only protect themselves, but also the rest of the non-vaccinated units of the community.

Unfortunately, this topic is often underestimated or even unknown among those who should be the first to be aware of it, in view of their professional role [18]. The study found that more than half the interviewed sample believed they knew this effect. (Table 1).

Notably, this was more prevalent among nurses with a specific education level.

Most people qualified with just a diploma claimed not to have knowledge of the effect while the majority of people with a Bachelor, a master or PhD claimed they knew about it. However, the data must be interpreted with caution because the questionnaire did not provide any further confirmation questions. Therefore, we can only assume that knowledge relating to immunity in the questionnaire is in fact as it appears from the data analysis. Further studies should be conducted regarding this matter.

In relation to the injection site of vaccines for children walking for less than a year or not walking yet, a higher percentage of nurses, paediatricians and radiologists answered accurately compared to students and pharmacists who answered incorrectly. (Table 2).

Within all the age ranges there was a high level of knowledge about the topic with high percentages of subjects giving the correct answer except for those in the over 61-year age range who answered incorrectly.

In conclusion, we noticed that there is a good level of information and knowledge about the injection site of vaccines for HCWs in all age groups, except for those over 61. In particular, the professional category of doctors was the most prepared compared to the others.

### Attitudes

As analysed in other studies [13], we observed that in all the professional categories the percentage in favour of vaccinations for their children was higher than 60%.

These data support the fact that the HCWs are the first to be aware of the importance of vaccination and its benefits against some diseases [17]. Unfortunately, the positive tendency of HCWs to vaccinate their own children does not necessarily equate to vaccinating themselves as revealed in previous studies [12, 20]. In our research, more then 50% of the people interviewed had not been vaccinated against influenza in the last 5 years. Only three people did not answer, therefore there was a high response to this question. (Table 3).

In the last 5 years, less than one third of the sample got vaccinated against influenza. That suggests that we need to encourage the vaccination campaign in the hospitals and raise awareness of its importance as much as possible.

In particular, among paediatricians and nurses the latter appear the category least inclined to get vaccinated.

Moreover, in the other professional categories, it must be noted that the majority do not get vaccinated even if the representative number is too limited to allow us to provide a meaningful statistic. (Table 3)

### Disinformation

Our results regarding vaccine information sources are similar to those from past research, which show a remarkable preference toward internet and paediatricians [21].

Data analysis shows that half the subjects interviewed acquired information on vaccinations from primary and secondary literature sources such as books and journals.

Half the sample uses the internet as a source of information and about 1/10 of the sample uses it as their only source.

Notably, more than 50% of nurses with a degree prefer to use the internet and paediatricians instead of scientific literature which is used by very few of them.

Among those with a bachelor’s degree a wide range of all available sources was used. (Table 4).

Generally, scientific sources were used infrequently. The higher the education level the more frequent the utilisation of trustworthy scientific resources and literature.

### Opinion

In addition to ensuring vaccination for their children, HCWs should be the first to provide and promote vaccination for their patients.

More than two thirds of the sample thought it really important, whereas a very small percentage believed it not to be so important or not important at all.

From the data obtained, we can state that the HCWs interviewed have a great sense of belonging to their job and a strong sensitivity towards the issue of vaccinations, as also highlighted in another study [16].

However, this result could also be attributed to the general sense of confidence in vaccines [18], that can derive from their studies. Indeed, more than half of the sample strongly agree that vaccines produce more benefits than risks.

Additional data in support of the sense of confidence in the effectiveness of vaccines is related to the use of alternative therapies to vaccination. In the study group a small percentage declared the use of this therapies [9, 11].

A small percentage of the use of medicine to increase immunity against influenza, as well as homeopathy and holistic disciplines are considered alternative therapies to vaccination.

These assumptions are connected with the category of nurses, particularly those who have a degree. (Table 5).

These data could be interpreted by considering the fact that before the introduction of a bachelor’s degree, the qualifying degree was strongly based on the strengthening of practical activities to the detriment of theory.

With the introduction of university courses for nurses over the years, the courses have endeavoured to increase the nurses’ knowledge in line with the growth of professional responsibility, whilst promoting the growth of medical and surgical clinical knowledge as well.

Moreover, the side effects connected to the influenza vaccination give cause for concern [12, 15]. Particularly, Guillan Barrè’s Syndrome, severe allergic reactions and the feeling of tiredness and fatigue resulting from vaccinations were the most cited in our study group. (Table 6).

The data also make us reflect on the actual perception of risk that may be lacking among some healthcare professionals.

In fact, half the sample believes that given their profession, the risk of contracting the disease is greater than for the general population.

In particular, half the nurses believe they have the same risk.

More than half the pharmacists and students consider they have a greater risk, while most of radiologists think they have the same risk.

In any case, given the general disinclination of HCWs to be vaccinated, they were asked what would the best way be for companies to propose influenza vaccination for them.

The majority suggested a written invitation (personal letter) to be vaccinated with information about the importance of vaccinations, indicating the place and times thereof.

A good percentage answered that it would be useful to give more visibility to the vaccination campaign using leaflets, posters and even thematic meetings. A lower percentage also mentioned the use of mandatory vaccinations for all healthcare personnel

## Conclusions

In line with the attitude observed in recent years among HCWs, the majority of HCWs at the “Anna Meyer” Children’s Hospital of Florence appeared not to be inclined to get vaccinated since around two thirds of the sample have not been vaccinated against the flu in the last 5 years. These data are particularly relevant in the category of nurses.

Nevertheless, healthcare professionals are in favour of vaccinating their children because they believe that vaccines offer more benefits than risks. At the same time, we found a good level of knowledge of HCWs in relation to vaccines and immunisation practices.

Consequently, this contradiction found in the results as well as low levels of adherence to flu vaccinations could not be connected to lack of knowledge, but rather, to a low perception of risks associated with flu by some HCWs.

Considering this results, greater efforts have to be made in communicating HCWs the decrease in the risk of being infected by flu if they are vaccinated. At this regard, it should be pointed out that flu is not a high-mortality disease, so that the results may be influenced by this. In this line, further research that analyses the level of vaccination for other more risky diseases is necessary.

In addition, health services should continue encouraging the increase of the knowledge and awareness of the utility of vaccines among their workers and also the society to prevent some diseases.

### Limitations of the study and future implications

The number of people interviewed largely covers the number required for statistical significance in the reference population, even if the number of representative subjects for students, pharmacists and radiologists is too small to draw any generalised conclusions for the above-mentioned categories.

The year of study of the students was not verified, so it is not possible to determine their knowledge of certain topics.

The peculiarities of the Meyer Children’s Hospital make it difficult to generalise the data for other healthcare settings differing from the paediatric one, and the sample should therefore be expanded with central investigations in other non-paediatric hospitals.

## Data Availability

All data generated or analysed during this study are included in this published article.

## Abbreviations

CHoF: Children’s Hospital of Florence
HCWs: Health care workers
PhD: Doctor of Philosophy
